# Translation and functional roles of circular RNAs in human cancer

**DOI:** 10.1186/s12943-020-1135-7

**Published:** 2020-02-15

**Authors:** Ming Lei, Guantao Zheng, Qianqian Ning, Junnian Zheng, Dong Dong

**Affiliations:** 1 Cancer Institute, Xuzhou Medical University, Xuzhou, Jiangsu China; 2 Center of Clinical Oncology, Affiliated Hospital of Xuzhou Medical University, Xuzhou, China

**Keywords:** Circular RNA, Translation, Function, Cancer, Biomarker

## Abstract

Circular RNAs (circRNAs) are a new class of non-coding RNAs formed by covalently closed loops through backsplicing. Recent methodologies have enabled in-depth characterization of circRNAs for identification and potential functions. CircRNAs play important roles in various biological functions as microRNA sponges, transcriptional regulators and combining with RNA binding proteins. Recent studies indicated that some cytoplasmic circRNAs can be effectively translated into detectable peptides, which enlightened us on the importance of circRNAs in cellular physiology function. Internal Ribosome Entry site (IRES)- and N^6^-methyladenosines (m^6^A)-mediated cap-independent translation initiation have been suggested to be potential mechanism for circRNA translation. To date, several translated circRNAs have been uncovered to play pivotal roles in human cancers. In this review, we introduced the properties and functions of circRNAs, and characterized the possible mechanism of translation initiation and complexity of the translation ability of circRNAs. We summarized the emerging functions of circRNA-encoded proteins in human cancer. The works on circRNA translation will open a hidden human proteome, and enhance us to understand the importance of circRNAs in human cancer, which has been poorly explored so far.

## Background

Circular RNAs (circRNAs) are a new type of endogenous RNAs produced by non-canonical back-splicing events [1, 2]. In circRNA, the downstream splice-donor site can be covalently linked to an upstream splice-acceptor site. The first circRNA molecule was discovered in an RNA virus in 1976 [3, 4], and circRNAs were observed in eukaryotic cell lines by electron microscopy in 1991 [5]. Thereafter, circRNAs have long been considered as aberrant splicing events [6]. Genomic and transcriptomic data generated by next generation sequencing projects and bioinformatics algorithms have identified significant amount of circRNAs in eukaryotes [7–14], clearly demonstrating that they are not merely the accidental byproducts or ‘splicing noise’.

High-throughput technologies have enabled in-depth characterization of circRNAs for identification and potential functions [15–17], such as ribosomal RNA depleted RNA sequencing, long-read sequencing and more importantly, improved circRNA mining bioinformatics algorithms. CircRNAs have been demonstrated as an abundant and conserved class of RNAs, and are widely expressed in a complex tissue-, cell type- or stage-specific manner [10, 18–22]. Recent studies confirmed the significant biological functions of circRNAs, especially in human cancers [23, 24]. CircRNAs can function as transcriptional regulators to control the expression of host genes [9, 25, 26]. They can also act as microRNA sponge to fine-tune the miRNA-mRNA regulatory axis [27–31]. It has been indicated that circRNA can serve as prognostic biomarkers because of their stable characteristics [32]. Moreover, several works have demonstrated hidden peptides encoded by circRNAs, which will largely broaden our understanding on the cellular physiology functions [33–35]. circRNAs have been considered as ‘non-coding’ elements, and the circRNA translation will provide a new perspective and new horizon for cancer treatment and diagnosis. We herein characterized the possible mechanism of translation initiation and complexity of the translation ability of circRNAs, and summarized the emerging functions of circRNA-encoded proteins in human cancers.

## Characterization of circRNAs

### Properties of circRNAs

CircRNAs are produced from precursor mRNA and are derived from canonical splice site. CircRNA biogenesis can compete with the maturation of its linear counterpart by linking 3′ splice site to a downstream 5′ splice site [25]. CircRNAs can be classified into three different types based on the type of sequence they contain: exonic circRNAs (EcRNA), intronic circRNAs (CiRNA) and exon-intron circRNAs (EIcRNA) [26, 36]. It has been documented that some intron-containing circRNAs are sequestered in the nucleus, while exonic circRNAs are exported to the cytoplasm. The currently discovered circRNAs are predominantly exonic and can be widely detected in the cytoplasma [28, 37].

The structures of circRNAs lack 5′ cap and 3′ end, which makes them resistant to the digestion of ribonucleases, such as RNase R, and confers a long half-life reaching up to 10 times that of linear RNAs [38]. Most circRNA sequences are highly conserved. Recent work demonstrated that approximately 15,000 human circRNA sequences can be detected in mouse or rat genomes [39, 40]. CircRNAs are generally expressed in vast majority of human tissues and are especially highly expressed in human brain [18, 40]. Furthermore, the expression of circRNAs always has a tissue- or cell- specific manner, which makes them a suitable candidate for biomarker studies in human cancer [10, 19, 20, 41].

### Genome-wide profiling of circRNAs

Ribosome RNA-depleted RNA-seq method has been widely employed for the discovery of novel circRNAs [2, 9, 16, 41–43]. This method can simultaneously provide the expression data for both coding and non-coding RNAs. Reliable quantification of circRNAs requires a substantial sequencing depth to ensure the identification of circRNAs. Salzman et al. identified “scrambled” exons and potential circRNA in human cancer using rRNA-depleted RNA-seq method [17]. In a recent work, Memczak et al. systematically explored circRNAs in human, mouse and nematode, and found that circRNAs are expressed in a tissue- and developmental stage-specific manner [29]. Subsequently, a genome-wide RNA exonuclease enrichment strategy was applied before sequencing. This method led to the enrichment of circRNAs in the sample, and a significantly higher number of circRNAs can be identified [9]. A recent work detected and characterized circRNAs across > 2000 cancer samples using exome capture RNA-seq, which achieved significantly better enrichment for circRNAs [44].

Bioinformatics methods for identifying novel circRNAs are constantly developed. They mainly identify circRNAs based on the presence of backsplice junction-spanning sequencing reads [16, 45, 46]. At first, sequencing reads that contiguously align to the genome and/or the transcriptome were filtered out, and those unaligned reads were used to align to the backsplice junctions. Sequencing errors and alignment mismatch can lead to false-positive identification [28, 46, 47]. These algorithms were developed to reduce false positives based on reads counts, number of samples having been detected, RNase R resistance, which inevitably results in the inability to detect some specific circRNA isoforms. In the absence of a clear gold-standard data, a combination of more than one circRNA prediction algorithms can largely minimize the false positives [48].

### Function of circRNAs

To date, the biological implications have only been investigated for a minor fraction of identified circRNAs, and most of them have been proposed to function as miRNA sponges in the cytoplasm [27–30, 49]. The most representative circRNA is ciRS-7, which contains more than 70 conserved binding sites for miR-7 [50–52]. In addition, circHIPK3 acts as a sponge for a variety of miRNAs to fine-tune transcriptional activity [53, 54]. Another important function of circRNAs is acting as protein sponge [25, 55]. Through binding of specific proteins to circRNAs, a molecular reservoir of proteins were created and act as decoys to facilitate a prompt response to extracellular stimuli.

## Translation of circRNAs

Based on our conventional opinions, 5′ and 3′ untranslated regions (UTR) are essential elements for the translation initiation in eukaryotic cells. Due to the absence of 5′ and 3′ ends, circRNAs were once considered as non-coding RNAs. Recently, mounting evidence have shown that circRNAs can be associated with polysomes and some of them comprise the initiation codon AUG and putative Open Reading Frames (ORF) with favorable length, which suggests an unexpected protein-coding potential for circRNAs [33, 56–58]. CircRNAs can actually encode regulatory peptides, and a hidden proteome encoded by circRNAs might exist.

In a first study, Chen and Sarnow suggested that engineered circRNAs in artificial constructs could recruit the 40s ribosomal subunit and initiate translation of detectable peptides in vitro [59]. However, this assay does not support the notion that circRNAs can also be translatable molecules in vivo. In 2015, Abe et al. demonstrated strong evidence concerning endogenous circRNAs serving as translation templates [56]. CircRNAs containing an infinite ORF can be efficiently translated in a rolling cycle amplification mechanism to produce a large protein concatemer. In 2017, Legnini et al. reported that *circ-ZNF609* is the backsplicing product of *ZNF609* exon 2, and can be translated into a protein in a splicing dependent and cap-independent manner in Myogenesis [60]. These works strongly supported the coding potential of endogenous circRNAs and raising the question how are circRNAs translated by non-canonical initiation mechanisms?

### Cap-independent translation initiation mechanisms of circRNAs

Eukaryotic mRNAs are always translated through the canonical cap-dependent translation. During the RNA synthesis process, a 7-methylguanosine cap is added to the 5′ end of mRNA for translation initiation. This structure can be organized by the eIF4E translation initiation factor, a protein complex including eIF4E, eIF4G and eIF4A components [61–63]. This well-known cap-dependent translation is the primary mode of translation initiation in eukaryotic cells. Under certain conditions, such as cellular stress or viral infection, an alternative mechanism known as cap-independent translation is employed to initiate mRNA translation through internal ribosome entry site (IRES) [64, 65]. IRESs are sequences located in the 5′ UTR of mRNAs that can directly recruit ribosomes to initiate translation. IRES-mediated translation was firstly identified in RNA and DNA viruses [66]. Subsequently, some works have reported that eukaryotic mRNAs can be also translated by IRES-mediated manner under stressful conditions [65, 67, 68], when canonical translation is altered, suggesting an alternative translation mechanism in eukaryotes to compensate for the defective cap-dependent translation. Furthermore, IRES-mediated translation has recently been indicated to play a key regulatory role during mammalian development. High-throughput screening for IRES elements in human genomes suggested that ~ 10% of the human mRNAs contain IRES elements [69, 70], however, the biological implications of IRES-mediated translation remain to be discovered. The translation of circRNAs can only be initiated by cap-independent fashion because of lacking 5′ cap and 3′ end. IRES-mediated fashion is one of the widely accepted mechanisms of circRNA translation initiation [35, 71, 72]. Recent works suggested that in vitro synthesized circRNAs in artificial constructs can be successfully translated, with an engineered IRES and split GFP sequences that can be joined together by precise back-splicing [73]. If the resulting circRNA can be translated from an IRES, a functional GFP protein can be successfully generated. To date, the number of IRESs in circRNAs is still unknown. A recent unbiased work using a cell-based reporter system identified a large number of AU-rich motifs (~ 10 nt) with IRES-like activity to initiate circRNA translation [74, 75]. Moreover, any circRNAs with the fragment longer than 50 nt is expected to contain an IRES-like hexamer by chance. The requirement of IRESs for circRNAs can be easily satisfied, suggesting the importance and prevalence of IRES-mediated cap independent translation mechanism of circRNAs in cytoplasm.

Another important cap-independent translation mechanism is through methylated adenosine residues in the form of N^6^-methyladenosines (m^6^A) in the 5′ UTR [76]. It has been shown that the m^6^A in the 5′ UTR can directly bind eukaryotic initiation factor 3 (eIF3) [76]. It is sufficient to recruit the 43S preinitiation complex and initiate translation bypass the m^7^G cap requirement, enabling a cap-independent mode of translation initiation. A recent work showed that 5′ URT m^6^A residues control ribosome scanning and subsequent start codon selection during integrated stress response [77]. The reinitiation of ATF4 mRNA is regulated by both the eIF2α signaling pathway and m6A modification in response to amino acid starvation. Interfering with the m^6^A demethylases and methyltransferases can influence ATF4 translation. So, 5′ UTR methylation in the form of m6A acts as a dynamic regulator in alternative translation [78]. The concept has been enlarged using m^6^A-seq method, and revealed that m6A in the 5′ UTR of other transcripts can also modulate start codon selection, thereby controlling alternative translation [77]. Using circRNA report genes, it has been reported that some short RNA elements containing m^6^A have IRES-like activity to initiate circRNA translation. Yang et al. showed that short RNA elements for the most abundant m^6^A were enriched in the circRNA sequences [79]. A single m^6^A modification is sufficient to initiate circRNA translation with the initiation factor eIF4G2 and the m^6^A reader protein YTHDF3. While depleting m^6^A demethylases FTO suppress circRNAs translation, knocking down m^6^A methyltransferases METTL3/14 promotes circRNA translation under the stress condition. High-throughput m^6^A-seq was performed with the RNase R treatment, indicating that at least 13% of circRNAs carry m^6^A modification. The authors identified 250 circRNAs to be associated with polysomes, which may have actively translation potential [79].

**Fig. 1:**
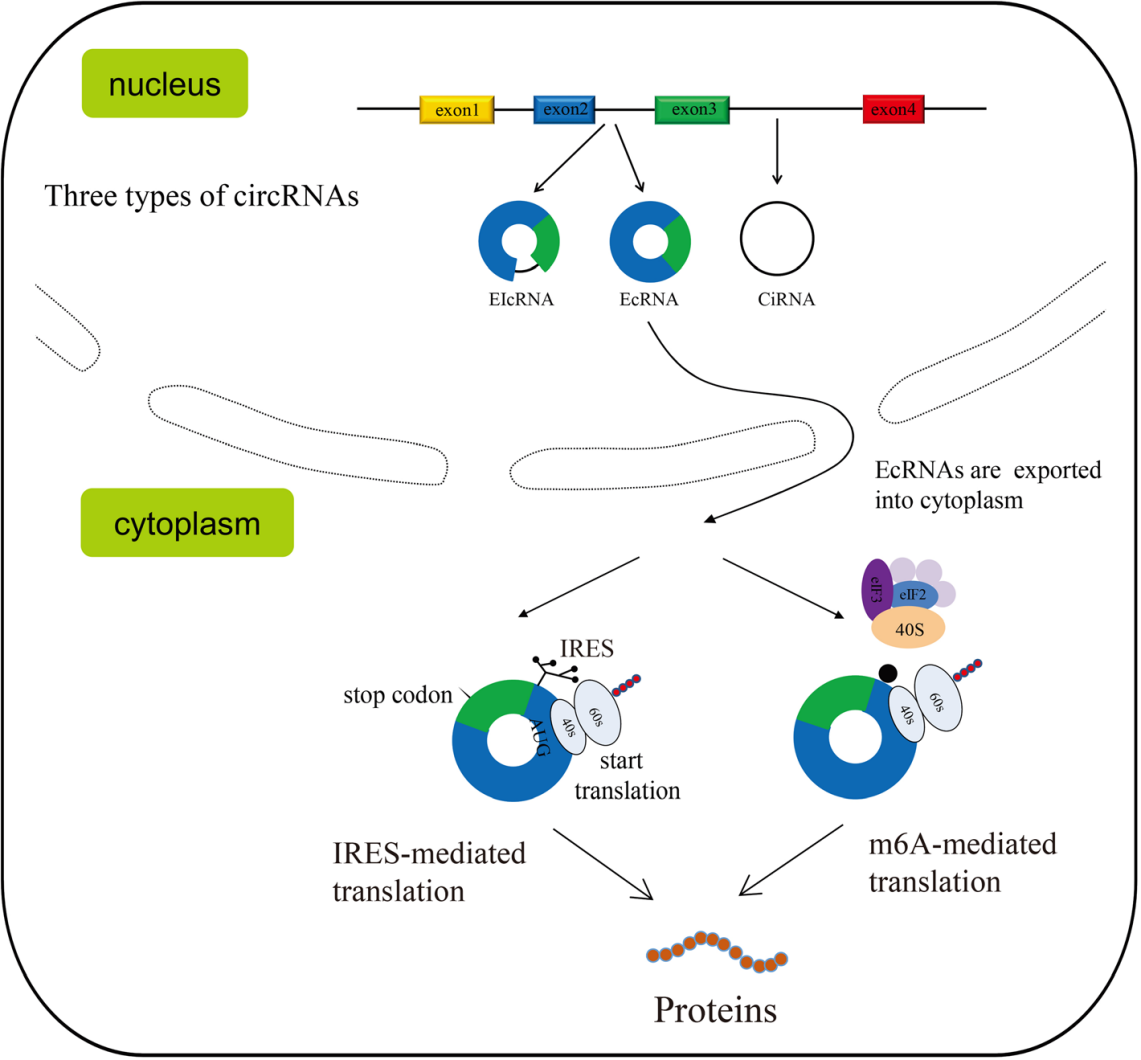
Cap-independent translation of circRNAs in eukaryotic cell. The circRNA can be divided into three different types: exonic (EcRNA), intronic (CiRNA) and exon-intron (EIcRNA) . CiRNAs and EIcRNAs are likely sequestered in the nucleus, while EcRNAs are mostly exported into cytoplasm. Internal Ribosome Entry site (IRES)- and N6-methyladenosines (m6A)-mediated cap-independent translation initiation are potential mechanisms for circRNA translation. These structures allow the internal recruitment of the 40S ribosomal subunit

Both IRES- and m6A-mediated translation initiation are important mechanisms for circRNA translation (Fig. 1). It is still unclear whether there is other cap-independent mechanism to initiate circRNA translation in eukaryotic cells.

### Bioinformatics tools for identifying circRNA coding potential

Systematic identification for circRNAs with coding potential using bioinformatics ways is necessary, and could provide insights into the function of circRNA-derived proteins. We herein summarized those bioinformatics tools for predicting circRNA coding potential **(**Table 1**)**. 1) Accurate and quantitative assessment of coding potential of circRNAs. ORF Finder is an easy-to-use graphical analysis tool to find all possible ORFs in the sequence provided by user [80]; CPC (Coding-Potential Calculator) is a widely used algorithm to assess the protein-coding potential of a transcript based on six biologically meaningful sequence features. The prediction of coding potential relied on sequence alignment using pairwise homology search for protein evidence [81]; PhyloCSF (Phylogenetic Codon Substitution Frequencies) determines whether the sequence is likely to represent a conserved protein-coding region using multiple alignments to calculate the phylogenetic conservation score [82]; CPAT (Coding-Potential Assessment Tool) is an alignment-free algorithm to distinguish between coding and noncoding transcripts on the basis of four sequence features using logistic regression [83]. A combination of these tools for the coding potential prediction can largely reduce the false positives. 2) IRES element identification. Sequences and structures of many IRES are well-known. IRESite is a database that can be used to examine the cellular internal ribosome entry sites [80]; CircInteractome database allow the investigation of potential circRNA translation through IRES sequences [84]; IRES finder is an improved computation method which can be used to perform a comprehensive search of IRES [85]. 3) feature analysis of circRNAs with coding potential. Pfam is a tool for the homology search of a putative product sequence [86]. The identification of a domain provides biological insights into its function. N-Glycosylation sites, Mucin-type O-glycosylation sites and phosphorylation sites can be predicted using NetNGlyc 1.0, NetOGlyc 3.1 and NetPhos 3.1 tools [87, 90], respectively. Some works built integrated bioinformatics tools for the identification of circRNAs with protein-coding potential, such as CircPro [88] and CircCode [89].

**Table 1:** Bioinformatics tools for identifying circRNA coding potential

Algorithms	Annotation	Refs
ORF Finder	This tool is to find all possible ORFs in the sequence provided by user	[80]
CPC	This tool is to assess the protein-coding potential of a transcript based on six biologically meaningful sequence features	[81]
PhyloCSF	This tool is to determine whether the sequence is likely to represent a conserved protein-coding region using multiple alignments to calculate the phylogenetic conservation score	[82]
CPAT	An alignment-free algorithm to distinguish between coding and noncoding transcripts on the basis of four sequence features	[83]
IRESite	A database that can be used to examine the cellular internal ribosome entry sites	[80]
CircInteractome	A database allow the investigation of potential circRNA translation through IRES sequences	[84]
IRES finder	An improved computation method which can be used to perform a comprehensive search of IRES	[85]
Pfam	A tool for the homology search of a putative product sequence	[86]
NetNGlyc 1.0	A tool for N-Glycosylation sites prediction	[87]
NetOGlyc 3.1	A tool for Mucin-type O-glycosylation sites prediction	[87]
NetPhos 3.1	A tool for phosphorylation sites prediction	[87]
CircPro	An integrated tool for the identification of circRNAs with protein-coding potential	[88]
CircCode	An integrated tool for the identification of circRNAs with protein-coding potential	[89]

### Experimental approaches for circRNA translation evaluation

After computational assessments, it is desirable to experimentally validate whether the circRNAs are indeed translatable. Ribosome profiling and polysomal fractionation enable translational global analysis. Ribosome profiling can capture footprints of actively translating ribosomes [91, 92]. From these footprints, we can infer the codon-by-codon movements of ribosomes. Recently, genome-wide translatomes were characterized using ribosome profiling in human heart [93]. A total of 40 ribosome bound circRNAs, produced from 39 genes, were identified across the backspliced junctions present in the ribosome footprints. Polysomal fractionation by sucrose density gradient centrifugation allows direct determination of translation efficiencies of circRNAs at a genome-wide scale [91, 94]. These two methods are complementary that enable genome-wide translational analysis. For those potential peptides encoded by circRNAs, mass spectrometry is a widely used platform for peptide detection [95].

### CircRNA translation in human cancer

To date, several translated circRNAs have been identified, which play pivotal roles in human cancers [96–101]. Here, we present each of them and their functions in human cancers **(**Table 2**)**.

**Table 2:** A list of circRNAs encoded in human cancers

Gene names	Cancer type	Encoded protein name	Cancer phenotype	Refs
*circSHPRH*	Glioma	SHPRH-146aa	The overexpression of SHPRH-146aa glioblastoma cells reduces their malignant behavior and tumorigenicity in vitro and in vivo	[96]
*circLINC-PINT*	Gliobalstoma	PINT87aa	It can suppress glioblastoma cell proliferation in vitro and in vivo	[98]
*circβ-catenin*	hepatocellular carcinoma	β-catenin-370aa	Silencing of circβ-catenin significantly suppresses malignant phenotypes in vitro and in vivo	[102]
*circFBXW7*	Glioma Breast cancer	FBXW7-185aa	Knockdown of FBXW7-185aa promoted malignant phenotypes in vitro and in vivo	[97] [103]
*circAKT3*	Glioblastoma	AKT3-174aa	Knockdown of circ-AKT3 enhanced the malignant phenotypes of astrocytoma cells	[101]
*circPPP1R12A*	Colon cancer	circPPP1R12A-73aa	circPPP1R12A played a critical role in proliferation, migration and invasion of colon cancer cells	[100]

#### Translation of circSHPRH in glioma

A new circRNA produced by the SNF2 histone linker PHD RING helicase (*SHPRH*) gene was identified [96]. Mature *circSHPRH* is formed by backsplicing of exons 26 to 29 and the total length of *circSHPRH* is 400 nucleotides. The motif of ‘UGAUGA’ contains the overlapping initiation and termination codon (initiation codon is AUG, and termination codon is UGA). The *circSHPRH* is the first circRNA having overlapping initiation and termination codon during protein translation in eukaryotic cells. *circSHPRH* encodes a novel 146 amino acids protein (SHPRH-146aa), and displayed a tumor suppressor activity during tumorigenesis. Further analysis suggests SHPRH-146aa protects SHPRH protein from degradation by the ubiquitin proteasome. Another recent studies indicated that *circSHPRH* was identified as a biomarker in hepatocellular carcinoma [104, 105]. It is therefore reasonable to speculate that *circSHPRH* has the potential to be translated in other human cancers.

#### Translation of circLINC-PINT in gliobalstoma

The *circLINC-PINT* is originated from the long intergenic non-coding RNA p53-induced transcript (*LINC-PINT*), and encodes a peptide with 87 amino acids (PINT87aa) [98]. This 1084 nt circRNA is formed by the circularization of exon 2. PINT87aa is concentrated in nucleus, and plays a tumor-suppressive role in the control of cell proliferation and tumorigenesis. PINT87aa can interact with PAF1 complex inhibiting the transcriptional elongation of multiple oncogenes. As the binding partner of PAF1 complex, PINT87aa plays important role in deciding PAF1 complex proper localization.

#### Translation of circβ-catenin in hepatocellular carcinoma (HCC)

The Wnt/β-catenin pathway has been extensively examined in HCC [99]. GSK3β-induced β-catenin phosphorylation and degradation can lead to the activation of Wnt pathway, which correlates with tumorigenesis and poor prognosis in HCC [102]. The *circβ-catenin* was generated by the backsplicing of exon 2 to 7 of the *CTNNB1* gene, forming a 1129 nt circRNA molecule. In circBase [106], a total of 24 circRNAs can be derived from *CTNNB1* gene, and *circβ-catenin* is the only isoform expressed in HCC. The *circβ-catenin* produces a 370 amino acid peptide (β-catenin-370aa), and mainly located in the cytoplasm. Further in vitro and in vivo assays indicated that *circβ-catenin* can promote HCC cell growth through activation of Wnt pathway. Acting as a decoy for GSK3β, β-catenin-370aa can lead to escape from GSK3β-induced β-catenin degradation.

#### Translation of circFBXW7 in glioma and breast cancer

A circRNA, named *circFBXW7*, was produced by the tumor suppressor E3 ligase FBXW7 [97]. The *circFBXW7* is derived from the backsplicing of exon3 to exon4 of the *FBXW7* gene with a length of 620 nt. The *circFBXW7* encodes a 185 amino acid peptide (FBXW7-185aa). FBXW7-185aa is thought to function as a tumor suppressor in glioma. It interacts competitively with the deubiquitinating enzyme USP28, which antagonizing USP28-induced c-Myc stabilization. Another recent work demonstrated that FBXW7-185aa can inhibit the proliferation and migration of triple-negative breast cancer cells by increasing the abundance of FBXW7 and inducing c-Myc degradation [103].

#### Translation of circPPP1R12A in colon cancer

Zheng et al. identified a new circRNA, named *circPPP1R12A*, produced by the *PPP1R12A* gene [100]. The *circPPP1R12A* have a 216 nt ORF to encode a 73 amino acid peptide (circPPP1R12A-73aa). Similar to *circSHPRH*, the circularization contains overlapping initiation and termination codons during protein translation. The expression of *circPPP1R12A* is significantly up-regulated in tumors, which displays a tumor suppressor activity in colon cancer. Functional analysis showed that CircPPP1R12A-73aa can promote the proliferation and metastasis abilities through activating Hippo-YAP signaling pathway in colon cancer.

#### Translation of circAKT3 in glioblastoma

Recently, Xia et al. characterized a circRNA, named circAKT3, produced by *AKT3* gene [101]. The *circAKT3* is generated from exon 3 to exon 7 of the *AKT3* gene with a full length of 524 nt, and encodes a 174 amino acid novel protein (AKT3-174aa). The analysis of *circAKT3* expression showed the potential tumor suppressor activity of AKT3-17aa. AKT3-174aa can interact with phosphorylated PDK1 and limits AKT3-thr308 phosphorylation as a molecular decoy, and plays a negative regulatory role in modulating the PI3K/AKT signal intensity.

## Future perspective

Although circRNAs have been discovered to be key regulators that mediate many fundamental cellular processes, they were once considered as non-coding because of the arbitrary site restriction. Despite being at an early stage, recent works have shown that circular RNAs play key roles in human cancers. The translation of circRNAs will open a hidden human proteome, and enhance us to understand the importance of circRNAs in human cancer, which has been poorly explored so far. It has been documented that some circRNAs exert their biological functions both through encoded peptides and by RNA-based regulatory mechanism. For example, *circ-SHPRH* can encode SHPRH-146aa peptide to suppress tumorigenesis in *glioma* [96], and it can function as miRNA sponge to inhibit HCC progression [104, 105]; circZNF609 can act as miRNA sponge to promote breast cancer progression [25], and it can encode protein in Myogenesis [60]. Some circRNAs are bi-functional in human cancer, and the bi-functional ability of circRNAs remains to be explored.

These reported circRNA encoded peptides have significant anti-tumor functions by interfering cancer metabolic reporgramming or metastasis. Therefore, circRNA-encoded peptides have great potential to become therapeutic target and tumor biomarkers. Given their high tumor specificity, circRNA encoded peptides will become a new resource for anti-tumor protein drug screen. To date, tumor biomarkers play important roles in early detection, precise treatment and prognosis prediction. Those circRNA encoded peptides have great potential to be useful tumor biomarkers. Taken together, the researches on circRNA encoded proteins will provide a new way for cancer diagnosis and therapy.

## Conclusion

New insights have been gained bout the translation of circRNAs and their functional roles in human cancer. In this review, we summarized the possible scenarios by which circRNAs can serve as translational template. A new perspective is opening for the research on circRNAs, and there’s no doubt that more and more new circRNA encoded proteins will be discovered in the near future. A hidden human proteome will enlighten us on the importance of circRNA in human cancer.

## Data Availability

Not applicable.

## Abbreviations

circRNAs: Circular RNAs
CiRNA: Intronic circRNAs
CPAT: Coding-potential assessment tool
CPC: Coding-potential calculator
EcRNA: Exonic circRNAs
EIcRNA: exon Intron circRNAs
HCC: Hepatocellular carcinoma
IRES: Internal ribosome entry site
m^6^A: N^6^-methyladenosines
ORF: Open reading frames
PhyloCSF: Phylogenetic codon substitution frequencies
UTR: Untranslated regions
